# The treatment outcomes and the use of adjuvant therapies in breast cancer patients with severe co-morbidities

**DOI:** 10.1371/journal.pone.0173721

**Published:** 2017-03-21

**Authors:** Jaihong Han, Han-Byoel Lee, Eun-Shin Lee, Young Joon Kang, Yumi Kim, Jihye Choi, Jiyoung Rhu, Hee-Chul Shin, Wonshik Han, Dong-Young Noh, Hyeong-Gon Moon

**Affiliations:** 1 Department of Surgery, Seoul National University College of Medicine, Seoul, Republic of Korea; 2 Department of Surgery, Chung-Ang University College of Medicine, Seoul, Republic of Korea; 3 Cancer Research Institute, Seoul National University College of Medicine, Seoul, Republic of Korea; University of North Carolina at Chapel Hill School of Medicine, UNITED STATES

## Abstract

**Purpose:**

Studies have suggested a potential role of patient’s co-morbidity in determining the survival outcomes of breast cancer. In this study, we examined the long-term oncologic outcomes in breast cancer patients who underwent curative surgery according to their pre-existing comorbid conditions and analyzed the association between the co-morbidity and the use of adjuvant therapies.

**Methods:**

The medical records of 2,501 patients who underwent surgery for primary breast cancer from June 2006 to June 2010 were reviewed retrospectively. The patients were classified into three groups according to preoperative ASA status determined by the anesthesiologists. Clinico-pathologic characteristics and survival outcomes of the patients were compared among the different co-morbidity groups.

**Results:**

There were 1,792 (71.6%), 665 (26.6%), and 44 (1.8%) patients in ASA I, II, and III, respectively. Total 95 (3.8%) deaths and 269 (10.8%) recurrences (loco-regional and distant) occurred during the median follow-up period of 71 months. Patients with high comorbidity showed significantly higher rate of deaths (51 (2.8%), 38 (5.7%) and 6 (13.6%) deaths in ASA I, II and III group, respectively, p<0.001). The ASA 3 patients also showed significantly higher rate of breast cancer recurrence when compared to other groups (180 (10.0%), 80 (12.0%) and 9 (20.5%) in ASA I, II, and III, respectively, p = 0.041). Significantly fewer patients in the high co-morbidity group received adjuvant therapies (77 (4.3%), 44 (6.6%) and 8 (18.2%) in ASA I, II, and III, respectively, p<0.001). The increased recurrence of breast cancer in the high morbidity group was mostly seen in patients who did not receive adjuvant therapies. The incidence of serious adverse effect during the adjuvant therapy did not differ according to the co-morbidity conditions.

**Conclusions:**

In this study, high comorbidity was related to increased risk of death and recurrence in breast cancer. The increased risk of recurrence in high co-morbidity group was mostly seen in patients who did not receive adjuvant therapies. Considering the relatively low rates of serious adverse effects in high co-morbidity patients who received adjuvant therapies, active use of adjuvant therapies in selected patients may improve survival outcomes in breast cancer patients with severe co-morbidities.

## Introduction

Breast cancer is the most common female malignancy and is the second common cause of cancer death in women.[1] Due to its high incidence, breast cancer is also a major health issue for women with medical co-morbidities. The presence of co-morbidities pose can influence the decision on the use of the various treatment modalities. Furthermore, several studies have reported a significant increase in cancer mortality and post-treatment morbidity in cancer patients with pre-existing health conditions.[2, 3]

Recent studies have suggested that the breast cancer patients who has significant co-morbidities tend to have increased treatment-related complication and deaths, and also carry higher risk for overall and cancer mortalities.[4–7] Researchers have suggested the lower rates of adjuvant therapy as well as the natural course of the pre-existing medical conditions was the potential link between the increased risk of death and the presence of co-morbidities.[8] However, some reported that elderly patients with co-morbidities have worse survival outcome despite the similar rates of adjuvant therapy administration.[9]

Population-based studies on the association of the co-morbidity and survival have advantages of less biased sample collection and large number of patients. However, they may carry weakness in identifying details of the individual patient’s adjuvant therapy information depending on the database they use.[10] In this study, we investigated the relationship between the breast cancer survival and the presence of co-morbidities in a relatively large cohort of patients treated in a single institution. We determined the degree of their co-morbidity by using the American Society of Anesthesiologists (ASA) classifications assessed at the time of the initial surgery.

## Materials and methods

### Patients and analysis

The medical records of 2,501 patients who underwent surgery for primary breast cancer from June 2006 to June 2010 at Seoul National University Hospital were retrospectively reviewed. The data of patients were obtained from the web-based database of Seoul National University Hospital Breast Care Center.[11]

We excluded patients with stage IV disease at the time of the diagnosis. Each patient’s pre-existing co-morbidities was assessed by using the ASA classification. Briefly, ASA I includes patients who are healthy, non-smoking, and minimal alcohol use at the time of surgery. ASA II includes patients with mild systemic disease such as well-controlled hypertension or diabetes. ASA III includes patients with severe systemic disease such as poorly controlled hypertension or diabetes, chronic obstructive pulmonary disease, and end-stage renal disease receiving regular dialysis. The ASA classification was determined at the preoperative interview with the anesthesiologists. Recurrence was defined as distant metastasis or loco-regional recurrence. The information of death date was obtained from the Korean National Statistical Office.

Survival outcome was estimated by the Kaplan-Meier method and compared across groups using the log-rank. For multivariate analysis, a Cox proportional hazards ratio model was used to estimate the adjusted hazard ratio for significance. All analyses were carried out using SPSS (version 19.0; SPSS Inc). The statistical significance was assumed at p < 0.05.

### Ethics and consent

This study approved by the Institutional Review Board of Seoul National University Hospital (IRB No. 1405-088-580). Written informed consents were taken prior to surgery in all patients to register their information in the database. The review and analysis of the collected information were separately approved. All procedures were done in accordance with the Declaration of Helsinki.

## Results

The characteristics of included patients are summarized in the Table 1. The median follow-up period of 2,501 analyzed patients was 71 months. There were 1,792 (71.6%), 665 (36.6%) and 44 (1.8%) patients in ASA I, II, and III groups, respectively. A total of 95 (3.8%) deaths, 72 breast cancer-related deaths (2.9%), and 269 (10.8%) recurrences (loco-regional recurrence and distant metastasis) occurred during the follow-up periods. Ninety-seven percent of the patients who received adjuvant chemotherapy completed the recommended cycles.

**Table 1:** Characteristics among 2,501 patients underwent surgery for breast cancer, 2006–2010.

Characteristics			Number (Percent)
Age			49.76 ± 10.14
Median follow-up period (month)			71.00
Gender	Male		6 (0.2%)
	Female		2495 (99.8%)
Operation type (breast)	Mastectomy		834 (33.3%)
	Breast conserving surgery		1667 (66.7%)
Operation type (axilla)	Axillary lymph node dissection		985 (39.4%)
	Sentinel lymph node biopsy		1414 (56.5%)
	Not done		102 (4.1%)
ASA^a^	1		1792 (71.6%)
	2		665 (26.6%)
	3		44 (1.8%)
Chemotherapy	Not done		940 (37.6%)
	Done		1555 (62.2%)
	Unknown		6 (0.2%)
Adjuvant Radiotherapy	Not done		714 (28.5%)
	Done		1774 (70.9%)
	Unknown		13 (0.5%)
Adjuvant hormone therapy	Not done		869 (34.7%)
	Done	Tamoxifen	1082 (43.2%)
		Aromatase inhibitor	517 (20.7%)
	Unknown		33 (1.3%)
Neoadjuvant chemotherapy	Not done		2210 (88.4%)
	Done		291 (11.6%)
Hormone receptor	Estrogen receptor	Positive	1637 (65.5%)
		Negative	853 (34.1%)
		Unknown	11 (0.4%)
	Progesterone receptor	Positive	1321 (52.8%)
		Negative	1169 (46.7%)
		Unknown	11 (0.4%)
	Her-2 (FISH)	Positive	520 (20.8%)
		Negative	1680 (67.1%)
		Unknown	301 (12.0%)
Subtype	HR^b^+HER-2-		1247 (49.9%)
	HR+HER-2+		218 (8.7%)
	HR-HER-2+		301 (12.0%)
	HR-HER-2-		433 (17.3%)
	Unknown		302 (12.1%)
Histologic grade	1		153 (6.1%)
	2		779 (31.1%)
	3		976 (39.0%)
	Unknown		593 (23.7%)
Event	Death		95 (3.8%)
	Recur (distant metastasis + loco-regional)		269 (10.8%)
	Breast cancer related death		72 (2.9%)
Stage	0^c^		314 (12.6%)
	I		890 (35.6%)
	II		928 (37.1%)
	III		369 (14.8%)
T stage	Tis		314 (12.6%)
	1		1061 (42.4%)
	2		937 (37.5%)
	3		158 (6.3%)
	4		31 (1.2%)
N stage	0		1617 (64.7%)
	1		577 (23.1%)
	2		204 (8.2%)
	3		103 (4.1%)

^a^ American Society of Anesthesiologists

^b^ Hormone receptor

^c^ Ductal carcinoma in situ

**Fig 1:**
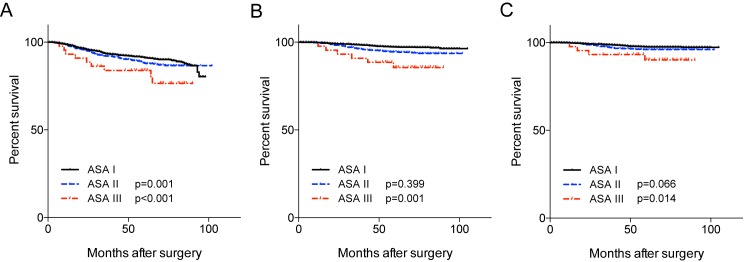
Disease-free survival, overall-survival, and breast cancer-specific survival according to the ASA status. Kaplan-Meier survival curves for the disease-free survival (A), overall survival (B), and breast cancer-specific survival (C) are shown (*P*-value is analyzed with comparing to ASA 1 group).

The presence of co-morbidities was significantly associated with various clinicopathologic variables as shown in the Table 2. Patients with ASA II or ASA III classification had more advanced tumors, were more likely to receive mastectomy, and were less likely to undergo adjuvant therapies with the exception of adjuvant hormonal therapy. There was no significant difference in the rates of patients who failed to complete the recommended cycles of chemotherapy according to the ASA groups. Survival analysis showed that the presence of co-morbidity had significant adverse effects on overall survival, breast cancer-specific survival, and breast cancer recurrence (Fig 1). In Cox multivariate regression analysis, the severity of co-morbidity was significantly associated with both overall survival and disease-free survival (Table 3)

**Table 2:** Characteristics in each American Society of Anesthesiologists (ASA) group.

ASA^a^			I	II	III	P-value
Patients			1792 (71.6%)	665 (26.6%)	44 (1.8%)	
Age			47.53 ± 9.18	54.94 ±10.19	62.27 ±9.04	<0.001
Follow-up period (month)			68.99 ± 17.89	65.75 ± 18.24	62.48 ± 19.76	<0.001
Gender	Male		2 (0.1%)	4 (0.6%)	0 (0.0%)	.083
	Female		1790 (99.9%)	661 (99.4%)	44 (100%)	
Operation type (breast)	Mastectomy		561 (31.3%)	250 (37.6%)	23 (52.3%)	<0.001
	Breast conserving surgery		1231 (68.7%)	415 (62.4%)	21 (47.7%)	
Operation type (axilla)	Axillary lymph node dissection		667 (37.2%)	295 (44.4%)	23 (52.3%)	.003
	Sentinel lymph node biopsy		1043 (58.2%)	352 (52.9%)	19 (43.2%)	
	Not done		82 (4.6%)	18 (2.7%)	2 (4.5%)	
Chemotherapy	Not done		649 (36.2%)	266 (40.0%)	25 (56.8%)	.018
	Done		1140 (63.6%)	396 (59.5%)	19 (43.2%)	
	Unknown		3 (0.2%)	3 (0.5%)	0 (0.0%)	
Adjuvant Radiotherapy	Not done		470 (26.2%)	223 (33.5%)	21 (47.7%)	<0.001
	Done		1314 (73.3%)	438 (65.9%)	22 (50.0%)	
	Unknown		8 (0.4%)	4 (0.6%)	1 (2.3%)	
Adjuvant hormone therapy	Not done		609 (34.0%)	245 (36.8%)	15 (34.1%)	<0.001
	Done	Tamoxifen	888 (49.6%)	185 (27.8%)	9 (20.5%)	
		Aromatase inhibitor	271 (15.1%)	227 (34.1%)	19 (43.2%)	
	Unknown		24 (1.3%)	8 (1.2%)	1 (2.3%)	
Neoadjuvant Chemotherapy	Not done		1605 (89.6%)	564 (84.8%)	41 (93.2%)	.003
	Done		187 (10.4%)	101 (15.2%)	3 (6.8%)	
Hormone receptor	Estrogen receptor	Positive	1181 (65.9%)	427 (64.2%)	29 (65.9%)	.936
		Negative	603 (33.6%)	235 (35.3%)	15 (34.1%)	
		Unknown	8 (0.4%)	3 (0.5%)	0 (0.0%)	
	Progesterone receptor	Positive	988 (55.1%)	310 (46.6%)	23 (52.3%)	.006
		Negative	796 (4.4%)	352 (52.9%)	21 (47.7%)	
		Unknown	8 (0.4%)	3 (0.5%)	0 (0.0%)	
	Her-2 (FISH)	Positive	354 (19.8%)	159 (23.9%)	7 (15.9%)	.111
		Negative	1226 (68.4%)	421 (63.3%)	33 (75.0%)	
		Unknown	212 (11.8%)	85 (12.8%)	4 (9.1%)	
Subtype	HR^b^+HER-2-		919 (51.3%)	302 (45.4%)	26 (49.9%)	.246
	HR+HER-2+		146 (8.1%)	70 (10.5%)	2 (4.5%)	
	HR-HER-2+		208 (11.6%)	88 (13.2%)	5 (11.4%)	
	HR-HER-2-		307 (17.1%)	119 (17.7%)	7 (15.9%)	
	Unknown		212 (11.8%)	86 (12.9%)	4 (9.1%)	
Histologic grade	1		108 (6.0%)	41 (6.2%)	4 (9.1%)	.801
	2		554 (30.9%)	210 (31.6%)	15 (34.1%)	
	3		691 (38.6%)	269 (40.5%)	16 (36.4%)	
	Unknown		439 (24.5%)	145 (21.8%)	9 (20.5%)	
Event	Death		51 (2.8%)	38 (5.7%)	6 (13.6%)	<0.001
	Recur (distant metastasis + loco-regional)		180 (10.0%)	80 (12.0%)	9 (20.5%)	.041
	Breast cancer related death		43 (2.4%)	25 (3.8%)	4 (9.1%)	.009
Stage	0^c^		237 (13.2%)	71 (10.7%)	6 (13.6%)	.004
	I		664 (37.1%)	214 (32.2%)	12 (27.3%)	
	II		655 (36.6%)	258 (38.8%)	15 (34.1%)	
	III		236 (13.2%)	122 (18.3%)	11 (25.0%)	
T stage	Tis		237 (13.2%)	71 (10.7%)	6 (13.6%)	.058
	1		781 (43.6%)	264 (39.7%)	16 (36.4%)	
	2		655 (36.6%)	263 (39.5%)	19 (43.2%)	
	3		101 (5.6%)	54 (8.1%)	3 (6.8%)	
	4		18 (1.0%)	13 (2.0%)	0 (0.0%)	
N stage	0		1191 (66.5%)	401 (60.3%)	25 (56.8%)	.019
	1		405 (22.6%)	161 (24.2%)	11 (25.0%)	
	2		134 (7.5%)	64 (9.6%)	6 (13.6%)	
	3		62 (3.5%)	39 (5.9%)	2 (4.5%)	

^a^ American Society of Anesthesiologists.

^b^ Hormone receptor.

^c^ Ductal carcinoma in situ.

**Table 3:** Multivariate and univariate analysis of mortality and recurrence in American Society of Anesthesiologists (ASA) groups.

		Overall survival								Cancer specific survival								Disease free survival							
		Univariate				Multivariate				Univariate				Multivariate				Univariate				Multivariate			
Prognostic factor		P	HR	95% C.I. for		P	HR	95% C.I. for		P	HR	95% C.I. for		P	HR	95% C.I. for		P	HR	95% C.I. for		P	HR	95% C.I. for	
				HR				HR				HR				HR				HR				HR	
Age		0.005	1.029	1.009	1.049	0.653	1.005	0.984	1.026	0.573	1.007	0.984	1.03					0.008	0.983	0.971	0.996	0.001	0.976	0.963	0.99
ASA^a^	I	0				0.005				0.011				0.003				0.015				0.002			
	II	0.001	2.084	1.369	3.173	0.096	1.471	0.933	2.318	0.074	1.567	0.957	2.565	0.399	1.241	0.751	2.051	0.066	1.281	0.984	1.669	0.077	1.29	0.973	1.709
	III	0	5.169	2.217	12.049	0.002	4.454	1.753	11.321	0.007	4.116	1.477	11.469	0.001	6.399	2.222	18.429	0.014	2.315	1.185	4.524	0.001	3.356	1.661	6.78
Neoadjuvant chemotherapy		0	4.701	3.099	7.133	0.307	1.39	0.739	2.618	0	6.19	3.897	9.833	0.058	1.931	0.978	3.811	0	3.218	2.459	4.21	0.024	1.58	1.063	2.349
Mastectomy		0	3.469	2.292	5.252	0.059	1.601	0.983	2.607	0	3.127	1.947	5.023	0.108	1.561	0.907	2.685	0	1.791	1.409	2.278	0.063	1.297	0.986	1.706
T stage	Tis	0				0.012												0				0.046			
	1	0.118	4.997	0.665	37.55	0.172	4.407	0.525	36.967	0				0.059				0.253	1.384	0.792	2.417	0.582	1.217	0.606	2.443
	2	0.007	15.232	2.1	110.499	0.051	8.582	0.995	74.004	0	3.81	1.878	7.731	0.396	1.396	0.646	3.02	0	3.05	1.787	5.206	0.128	1.782	0.847	3.75
	3	0	54.575	7.395	402.782	0.012	17.698	1.899	164.896	0	17.047	8.113	35.821	0.017	3.208	1.232	8.354	0	7.182	4.002	12.886	0.024	2.619	1.133	6.053
	4	0	83.319	10.249	677.318	0.022	15.849	1.494	168.118	0	24.283	8.823	66.831	0.125	2.691	0.76	9.523	0	9.005	4.039	20.074	0.097	2.413	0.854	6.819
N stage	0	0				0				0				0				0				0.003			
	1	0.013	2.043	1.165	3.582	0.193	1.542	0.804	2.958	0.019	2.3	1.15	4.599	0.45	1.345	0.624	2.901	0.002	1.582	1.175	2.13	0.753	1.059	0.742	1.512
	2	0	6.616	3.827	11.437	0.001	3.373	1.681	6.767	0	9.75	5.151	18.457	0	4.142	1.874	9.154	0	3.359	2.398	4.705	0.02	1.661	1.083	2.549
	3	0	13.234	7.602	23.039	0	4.484	2.127	9.454	0	14.899	7.526	29.495	0.002	3.916	1.65	9.29	0	5.451	3.708	8.014	0.001	2.248	1.376	3.671
HG	1	0				0.004				0				0.006				0				0			
	2	0.287	3.001	0.396	22.718	0.495	2.034	0.264	15.645	0.575	1.807	0.229	14.262	0.966	1.046	0.13	8.409	0.109	2.119	0.846	5.304	0.247	1.728	0.685	4.359
	3	0.016	11.443	1.589	82.393	0.116	5.078	0.669	38.556	0.027	9.305	1.288	67.216	0.266	3.165	0.415	24.144	0	6.049	2.486	14.719	0.003	4.004	1.592	10.068
Subtype	HR^b^+/HER-2-	0				0.018				0				0.055				0				0.564			
	HR+/HER-2+	0.497	0.661	0.2	2.184	0.195	0.449	0.134	1.505	0.655	0.717	0.166	3.091	0.205	0.385	0.088	1.686	0.02	1.644	1.082	2.499	0.598	1.123	0.729	1.731
	HR-/HER-2+	0.001	2.858	1.551	5.268	0.989	0.993	0.385	2.56	0	3.794	1.912	7.528	0.837	1.124	0.367	3.449	0	2.246	1.597	3.158	0.356	1.303	0.743	2.285
	HR-/HER-2-	0	5.198	3.201	8.442	0.067	2.246	0.944	5.344	0	5.846	3.32	10.294	0.127	2.258	0.793	6.433	0	2.417	1.796	3.254	0.202	1.423	0.828	2.447
Chemotherapy		0.021	1.724	1.084	2.741	0	0.313	0.173	0.566	0.058	1.79	0.982	3.263					0	2.191	1.639	2.931	0.043	0.654	0.433	0.987

^a^ American Society of Anesthesiologists.

^b^ Hormone receptor.

**Fig 2:**
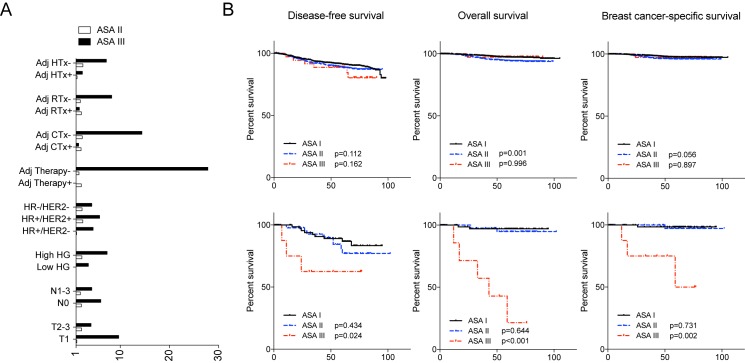
Survival of patients according to their ASA status and the use of adjuvant therapies. The relative risks of recurrence in ASA II and ASA III patients compared to ASA I in various subgroups of studied patients are shown in Fig 2A. Survival outcomes of patients who received adjuvant therapies (B, upper panels) and who did not receive adjuvant therapies (B, lower panels).

We tested the prognostic impact of co-morbidity according to the various clinical and pathologic factors. The increased hazard ratio of the severe co-morbidity was most evident in patients who did not receive adjuvant therapies (Fig 2A). Indeed, the patients with ASA III classification showed significantly worse outcome in tumor recurrence and deaths when they did not receive adjuvant therapies (Fig 2B). This prognostic association was not seen in patients who underwent adjuvant therapies. We further investigated the incidence of serious adverse effect during adjuvant therapy in 2,367 patients who underwent adjuvant therapy including 36 patients in ASA III classification. The incidence of grade 3 and 4 adverse effects was not significantly different according to the ASA classification.

## Discussion

In the present study, we show that breast cancer patients with pre-existing co-morbidities have higher risk of recurrences and deaths by using the clinical data from 2,501 operable breast cancer patients treated in single institution. The significant association between the presence of co-morbidity and the poor treatment outcome was mostly seen in patients who did not receive postoperative adjuvant therapies. Our results suggest that conservative administration of adjuvant therapies in these patients may have resulted in the increased rate of recurrences and deaths.

Our results are in line with several previous observations on the relationship between the co-morbidity and survival in many cancer types including breast cancer.[12, 13] These studies have consistently shown that women with significant pre-existing co-morbidity have shorter overall survival. However, there are conflicting results on the association between the presence of co-morbidity and the risk of recurrence or metastasis. Based on the cancer registry data in Denmark, Land et al [10] have reported that the patients with higher degree of co-morbidity had significantly lower rates of recurrences despite their increased risk of deaths. Gold et al [14], on the other hand, have shown that omission of appropriate adjuvant therapies can wosen the treatment outcome even in early breast cancer patients. Our data adds evidence to the increased risk of recurrence in patients with significant co-morbidities.

It is a challenging issue for clinicians to determine the optimal treatment approaches for breast cancer patients with severe co-morbidities. For surgical treatment, axillary node dissection or nodal staging is often minimized in elderly patients with co-morbidities. On the other hand, mastectomy over breast conservation is often recommended to minimize the need for the adjuvant radiation to the chest wall. We also observed a significantly higher rate of mastectomy in patients with severe co-morbidities. Patient’s pre-existing co-morbidities and their functional status also influence the decision of adjuvant systemic therapies.[15] While it is rational to omit systemic therapy in patients with severe co-morbidity who are vulnerable to serious adverse effects, it also raises oncologic concerns since the adjuvant cytotoxic therapies can reduce the rate of recurrence and mortality in elderly breast cancer patients.[16] In our data, the significant association between the co-morbidity and increased risk of recurrence was only seen in patients who did not receive adjuvant therapies suggesting that omitting adjuvant therapy in patients with severe co-morbidity can result in disease recurrences. Additionally, we did not observe an increased risk of developing serious adverse effects in patients with severe co-morbidity who underwent adjuvant therapies. Similary to our observation, Klepin et al [17] reported no difference in treatment-related toxicities according to the co-morbidity status in patients undergoing cytotoxic chemotherapy.

Our study carries several important limitations. We used ASA classification and did not adopt the Charlson Comorbidity Index which is a commonly-used tool in assessing the degree of co-morbidity. However, ASA classification is well-correlated with Charlson Comorbidity Index and it has been shown to predict treatment-related outcomes.[18] Another limitation is the small number of ASA II and III patients that may weaken the present findings. Finally, a retrospective nature of this study warrants a prospective and multicenter observational study to validate the association between the patient’s co-morbidity status and oncologic outcomes.

## Conclusion

In this study, high comorbidity was related to higher rates of mortality and recurrence in breast cancer patients. Especially, in high comorbidity patients group who did not receive adjuvant therapy, the rate of mortality and recurrence was higher. Considering the relatively low rates of serious adverse effects in high co-morbidity patients who received adjuvant therapies, active use of adjuvant therapies in selected patients may improve survival outcomes in breast cancer patients with severe co-morbidities.

## Supporting information

S1 File The basic clinical datasets used for the analysis. The S1 file.xls contains information of co-morbidity and survival of the studied patients.
(XLSX)
